# *Eucalyptus camaldulensis* leaf extract enhances thalidomide efficacy in acute myeloid leukemia cells through modulation of mitochondrial energetics

**DOI:** 10.1186/s12906-026-05503-2

**Published:** 2026-08-13

**Authors:** Samah E. Ismail, Ashraf A. Hassan, Amany A. Elkhedel, Amal M. Fakhry, Eman A. Khalifa, Soliman M. Toto

**Affiliations:** 1 Applied Medical Chemistry Department, Medical Research Institute, Alexandria University, Alexandria, Egypt; 2 Botany and Microbiology Department, Faculty of Science, Alexandria University, Alexandria, Egypt; 3 Histochemistry and Cell Biology Department, Medical Research Institute, Alexandria University, Alexandria, Egypt

**Keywords:** Eucalyptus camaldulensis, Acute myeloid leukemia, Oxidative phosphorylation, Thalidomide, Mitochondrial dynamics

## Abstract

**Background:**

Acute myeloid leukemia (AML) is an aggressive hematological malignancy characterized by high relapse rates and poor prognosis. Leukemic stem cells (LSCs), a major driver of therapy resistance, rely predominantly on mitochondrial oxidative phosphorylation (OXPHOS) for survival. Targeting mitochondrial metabolism represents a promising therapeutic strategy. This study investigated the effect of *Eucalyptus camaldulensis* Dehnh. leaf extract, alone and in combination with thalidomide, on mitochondrial energetics in AML cells.

**Methods:**

An in vitro study was conducted using the human AML Kasumi-1 cell line. Cells were divided into four groups: control, thalidomide-treated, *E. camaldulensis* extract-treated, and combination-treated groups. The ethanolic extract was characterized by GC–MS and HPLC. Cell viability was assessed using WST-1 assay and IC₅₀ values were calculated. Gene expression of CD34, MFN1, MFN2, OPA1, and IL-6 was quantified by qRT-PCR. ATP levels were measured by ELISA. Mitochondrial length was assessed by transmission electron microscopy.

**Results:**

*E. camaldulensis* extract significantly reduced Kasumi-1 cell viability in a concentration-dependent manner, both alone and in combination with thalidomide. The combination demonstrated an additive cytotoxic effect (CI = 1). The IC₅₀ values were 197.80 µg/mL for thalidomide, 30.26 µg/mL for the extract, and 28.26 µg/mL for the combination. Thalidomide alone or combined significantly downregulated IL-6 expression, while thalidomide increased MFN1 expression. The extract significantly downregulated IL-6 and mitochondrial fusion genes and reduced CD34 expression. Extract treatment decreased ATP levels and shortened mitochondrial length compared with untreated cells (*p* < 0.05).

**Conclusion:**

Eucalyptus camaldulensis extract disrupts mitochondrial energetics and LSC-associated pathways in AML cells. Its combination with thalidomide shows additive activity and may represent a complementary strategy to overcome metabolic-driven therapy resistance in AML.

## Background

Acute myeloid leukemia (AML) is the most common acute leukemia in adults and accounts for approximately 80% of acute leukemia cases [1]. Despite advances in therapeutic strategies, AML remains associated with poor prognosis and a high relapse rate, largely due to primary and acquired drug resistance [2].

Accumulating evidence indicates that leukemic stem cells (LSCs) are central drivers of therapy resistance and disease recurrence in AML. Unlike normal hematopoietic stem cells (HSCs), which primarily rely on glycolysis for ATP production, LSCs exhibit a distinct metabolic phenotype characterized by dependence on mitochondrial oxidative phosphorylation (OXPHOS) for energy production and resistance to cancer therapy [3–5]. This metabolic reprogramming enhances ATP generation, supporting drug efflux through ATP-binding cassette transporters and promoting chemotherapy resistance and relapse [6]. Therefore, targeting OXPHOS represents a promising strategy to selectively eradicate LSCs and overcome metabolic-driven drug resistance.

Mitochondrial dynamics play a crucial role in maintaining mitochondrial respiration and cellular metabolism. Mitochondrial fusion is mediated by three dynamin-related GTPases: mitofusin-1 (MFN1) and mitofusin-2 (MFN2), located on the outer mitochondrial membrane, and optic atrophy-1 (OPA1), located on the inner mitochondrial membrane [7]. Disruption of mitochondrial fusion has been shown to impair OXPHOS activity and induce cell cycle arrest at the G1/S transition in AML cells, nominating mitochondrial fusion regulators as potential therapeutic targets [8].

CD34, a transmembrane glycoprotein expressed on hematopoietic stem and progenitor cells, is widely used as a marker to identify and characterize LSCs in AML [9, 10]. Elevated CD34 expression has been associated with poor clinical outcomes and resistance to conventional therapies, further highlighting the importance of targeting LSC-associated pathways.

Given the urgent need for novel therapeutic strategies, immunomodulatory drugs (IMiDs) have gained attention as potential monotherapies or adjuncts to standard induction regimens in AML [11]. Thalidomide, a prototypical IMiD, exerts anti-inflammatory and immunomodulatory effects, including selective inhibition of interleukin-6 (IL-6) and tumor necrosis factor-alpha (TNF-α), both implicated in AML progression and survival signaling [12]. However, the impact of thalidomide on mitochondrial metabolism and OXPHOS in AML remains insufficiently explored.

Natural products have long been investigated as complementary therapeutic agents in cancer management [13]. *Eucalyptus camaldulensis* Dehnh. (Myrtaceae) is a widely used medicinal plant known for anti-inflammatory, antioxidant, and immunomodulatory properties. Previous studies have demonstrated that *E. camaldulensis* extracts exhibit significant antioxidant activity and anti-proliferative effects in various cancer cell lines [14–16]. Nevertheless, its potential role in modulating mitochondrial metabolism and OXPHOS in AML has not yet been elucidated.

Therefore, the present study aimed to investigate the effects of thalidomide and *E. camaldulensis* leaf extract, individually and in combination, on mitochondrial energetics, and leukemic stem cell-associated marker in AML cells.

## Materials and methods

### Study design and ethical approval

This in vitro experimental study was conducted using the human acute myeloid leukemia Kasumi-1 cell line as the malignant cellular model. The study protocol was approved by the Ethical Committee of the Medical Research Institute, Alexandria University (Approval No. E/C. S/N. T60/2024).

Kasumi-1 cells were allocated into four experimental groups according to the treatment regimen: (I) untreated control (drug-free medium); (II) thalidomide-treated; (III) *E. camaldulensis* extract-treated; and (IV) combination-treated (thalidomide + *E. camaldulensis* extract).

### Preparation of treatments

Thalidomide: A stock solution of thalidomide (50 mg/mL) was prepared by dissolving a Thalix-100 capsule (Each capsule contains Thalidomide USP 100 mg and capsule shell contains Titanium Dioxide) (Fresenius Kabi India Pvt Ltd) in 2 mL dimethyl sulfoxide (DMSO). The stock was diluted in complete culture medium to obtain working concentrations ranging from 0.1 to 1000 µg/mL.

Collection and preparation of *Eucalyptus camaldulensis* extract: Leaves of *E. camaldulensis* were collected in February 2024 from the Public Garden, Ismailia, Egypt (30°37’00.6"N, 32°15’54.0"E). Botanical identification was performed by Prof. Amal M. Fakhry (Department of Botany and Microbiology, Alexandria University). A voucher specimen (No. 4117) was deposited at the Herbarium of Alexandria University (ALEX). Leaves were washed, air-dried, and pulverized. A total of 300 g dry powder was extracted sequentially with 70% ethanol (three cycles, three days each). The filtrate was concentrated under reduced pressure at 60 °C using a rotary evaporator and lyophilized to yield 66 g of dry extract. The extract was stored at 4 °C until use. A stock solution was prepared and diluted in complete medium to obtain working concentrations ranging from 0.1 to 100 µg/mL.

### Phytochemical characterization

GC–MS analysis: The chemical composition of the extract was analyzed using a Trace GC1310-ISQ mass spectrometer (Thermo Scientific, USA) equipped with a TG-SMS capillary column (30 m × 0.25 mm × 0.25 μm). Helium was used as the carrier gas at 1 mL/min. The oven temperature was programmed from 50 °C (held) then increased by 5 °C/min to 230 °C (held 2 min), then increased to 290 °C by 30 °C/min (held 2 min). Injector and transfer line temperatures were 250 °C and 260 °C, respectively. Electron impact spectra were collected at 70 eV over m/z 40–1000 [17]. Compounds were identified by comparison with WILEY 09 and NIST 11 libraries.

HPLC analysis: HPLC analysis was performed using an Agilent 1100 system equipped with a UV/Vis detector and a Nucleosil^®^ C18 column (25 mm × 3.2 mm, 5 μm). Phenolic acids were separated using methanol (A) and acetic acid in water (1:25) (B) with a gradient: 100% B for 5 min, 50% A for 10 min, 80% A for 10 min, then 50% A for 5 min [18]. Flavonoids were separated using methanol: water (50:50, v/v) adjusted to pH 2.8 with phosphoric acid at 1.0 mL/min [19]. Alkaloids were separated using acetonitrile: methanol: orthophosphoric acid (55:45:1) at 1.0 mL/min with detection at 280 nm [20]. Terpenoids were separated using methanol:0.1% formic acid (92:8, v/v) at 0.4 mL/min with detection at 210 nm [21]. Tannins were separated using methanol: water (50:50, v/v) at 1.0 mL/min with detection at 280 nm. Chromatograms were analyzed using Agilent ChemStation [22].

### Cell culture

All cell culture assays were performed under strictly aseptic conditions. Kasumi-1 cells (ATCC, USA) were maintained in RPMI-1640 medium supplemented with 10% heat-inactivated fetal bovine serum (FBS) and 1% penicillin/streptomycin. Cells were cultured at 37 °C in a humidified incubator with 5% CO₂ and 95% air [23].

### Cell viability assay (WST-1)

Cell viability was assessed using the WST-1 assay (Abcam^®^, ab155902). Aliquots of 50 µL cell suspension (3 × 10³ cells) were seeded into 96-well plates and incubated for 24 h. Cells were then treated for 48 h with 50 µL medium containing thalidomide (0.1, 1, 10, 100, 1000 µg/mL), *E. camaldulensis* extract (0.1, 1, 10, 100 µg/mL), or combination treatment at the indicated concentrations, in triplicate. After 48 h, 10 µL WST-1 reagent was added and absorbance was measured after 1 h at 450 nm using a TECAN Infinite F50 microplate reader. Percent viability was calculated as treated/untreated absorbance × 100.

IC₅₀ values were determined using SigmaPlot. Drug interaction was evaluated using the Chou–Talalay method: CI < 1 indicates synergy, CI = 1 additive effect, and CI > 1 antagonism.

### Gene expression analysis (qRT-PCR)

Kasumi-1 cells were seeded in 6-well plates at 3 × 10⁵ cells/well and incubated for 24 h. Cells were treated with IC₅₀ concentrations of thalidomide and/or extract for 48 h in triplicate. Total RNA was extracted using RNeasy Mini Kit (Qiagen, Germany). qRT-PCR was performed using Rotor-Gene Q system (Qiagen) and A.B.T.™ 2X qRT-PCR Mix (SYBR) Low ROX kit (Applied Biotechnology, Egypt). Expression of CD34, MFN1, MFN2, OPA1, and IL-6 was quantified. GAPDH served as the reference gene (QuantiTect Primer Assay, Qiagen). Relative expression was calculated using the 2 − ΔΔCt method. Primer sequences for target genes are provided in Table 1.

**Table 1:** Primer sequence for target genes

Gene	Accession Number	Primer Sequence
CD34	NM_001773.3	Forward: 5’CCTCAGTGTCTACTGCTGGTCT3’
		Reverse: 5’GGAATAGCTCTGGTGGCTTGCA3’
MFN1	NM_033540.3	Forward: 5’GGTGAATGAGCGGCTTTCCAAG3’
		Reverse: 5’CCTCCACCAAGAAATGCAGGC3’
MFN2	NM_014874.4	Forward: 5’ATTGCAGAGGCGGTTCGACTCA3’
		Reverse: 5’TTCAGTCGGTCTTGCCGCTCTT3’
OPA1	NM_001354664.2	Forward: 5’GTGGTTGGAGATCAGAGTGCTG3’
		Reverse: 5’GAGGACCTTCACTCAGAGTCAC3’
IL-6	NM_000600.5	Forward: 5’AGACAGCCACTCACCTCTTCAG3’
		Reverse: 5’TTCTGCCAGTGCCTCTTTGCTG3’

### ATP measurement (ELISA)

Cells were treated as described for gene expression analysis. Pellets were resuspended in 0.1 M PBS (pH 7.2) to obtain 1 × 10⁶ cells/mL. ATP levels were measured using Human Adenosine triphosphate ELISA Kit (Cat. No. E3132Hu, Bioassay Technology Laboratory, China) following the manufacturer’s instructions. Concentrations were calculated from a standard curve at 450 nm.

### Transmission electron microscopy (TEM)

After 48 h treatment with IC₅₀ concentrations, cell pellets were fixed in 2.5% glutaraldehyde in 0.1 M phosphate-buffered saline (pH 7.2) at 4 °C for 3 h, washed, postfixed in 2% osmium tetroxide at 4 °C for 2 h, dehydrated in graded acetone, embedded in resin, and polymerized at 60 °C for 2 days. Ultrathin Sect.  (60 nm) were cut using a Leica Ultracut R ultramicrotome, stained with 1% aqueous uranyl acetate (5 min) and 0.4% lead citrate (2 min), and examined using a JEOL JSM-1400 Plus TEM. For each group, 20 mitochondria were randomly selected at ×8000 magnification and mitochondrial lengths were measured.

### Statistical analysis

Data were analyzed using IBM SPSS version 20.0. Normality was assessed using the Shapiro–Wilk test. Quantitative data were expressed as mean ± SD. Comparisons between two groups were performed using Student’s t test. A p value ≤ 0.05 was considered statistically significant. All experiments were performed in triplicate (*n* = 3).

## Results

### GC–MS analysis of *E. camaldulensis* extract

**Fig. 1:**
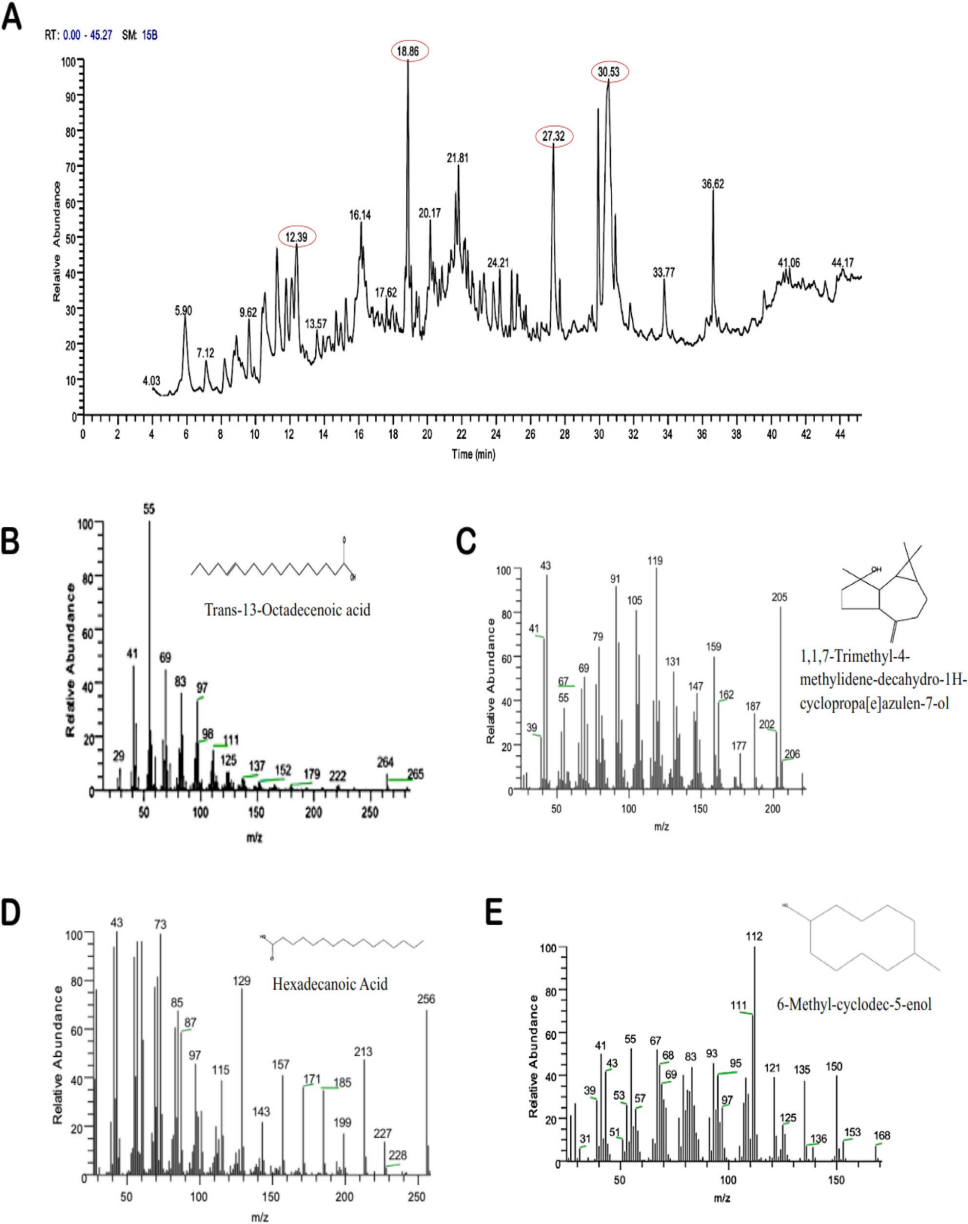
GC–MS chromatographic analysis of *Eucalyptus camaldulensis* ethanolic leaf extract. **A** Total ion chromatogram showing retention times of detected bioactive compounds. **B**–**E** Mass spectra of dominant compounds including trans-13-octadecenoic acid, 1,1,7-trimethyl-4-methylidene-decahydro-1 H-cyclopropa[e]azulen-7-ol, hexadecanoic acid, and 6-methyl-cyclodec-5-enol

The total ion chromatogram of the ethanolic leaf extract of *E. camaldulensis* is shown in Fig. 1, demonstrating retention times of detected constituents. The detailed chemical composition of identified compounds is presented in Table 2.

**Table 2:**
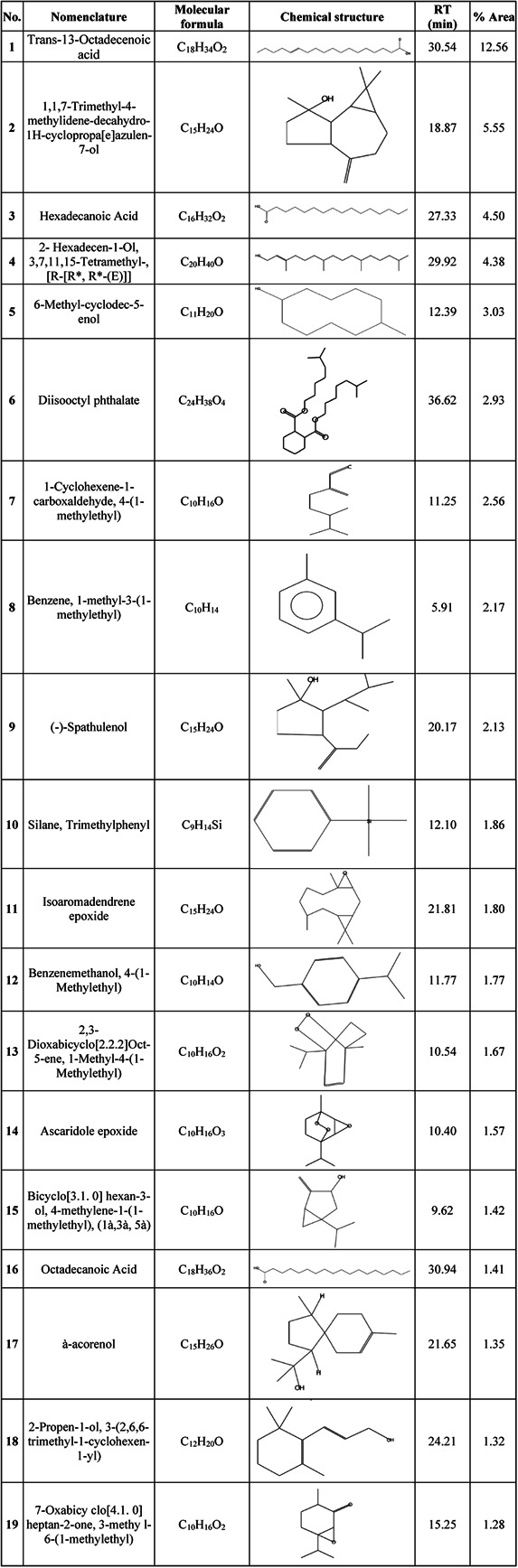
GC-MS analysis of bioactive compounds in *E*. *camaldulensis* ethanolic extract

### HPLC analysis of *E. camaldulensis* extract

**Fig. 2:**
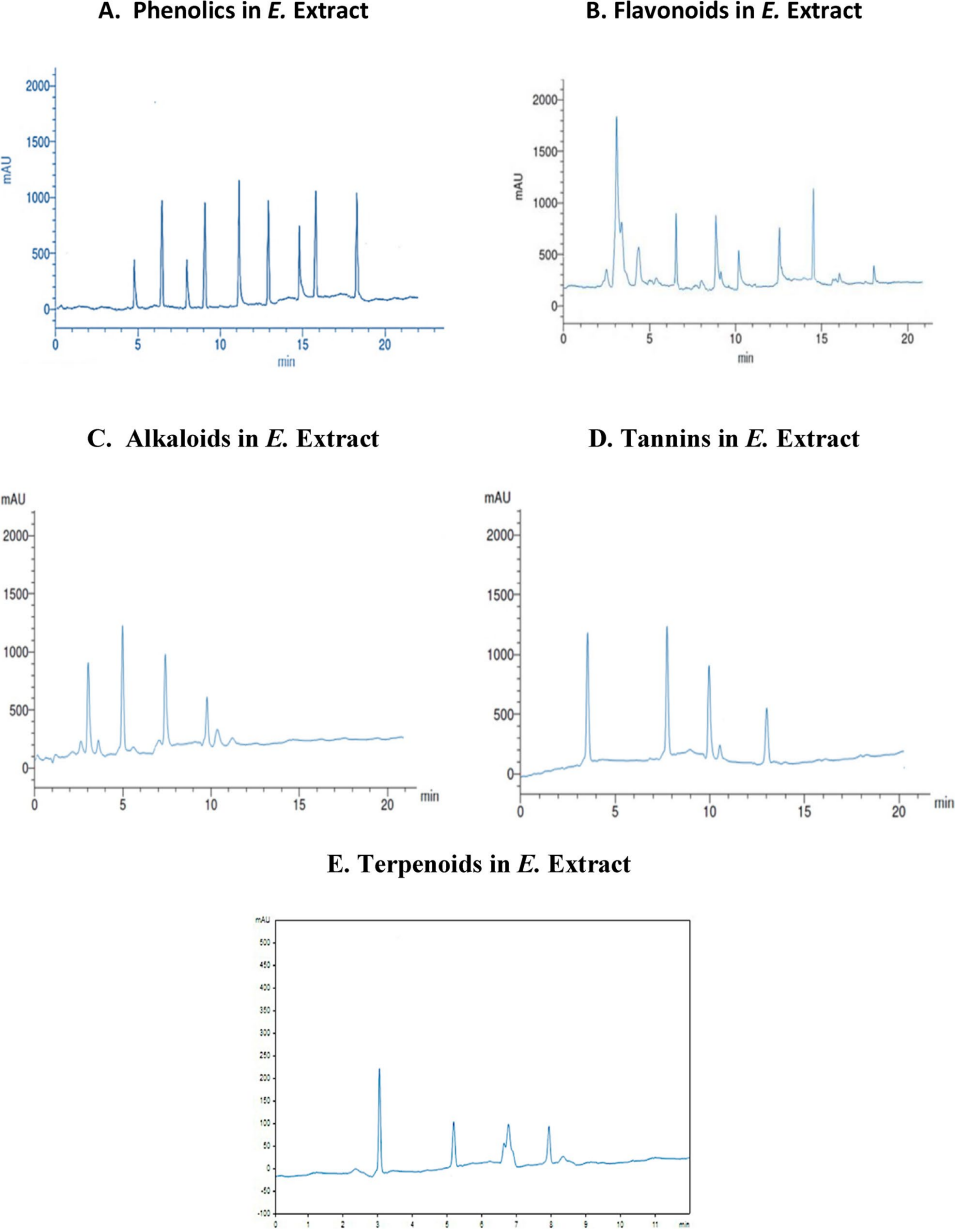
HPLC chromatographic profiling of *Eucalyptus camaldulensis* ethanolic extract. **A** Phenolic compounds; (**B**) Flavonoids; (**C**) Alkaloids; (**D**) Tannins; (**E**) Terpenoids

HPLC fractionation revealed that the extract contained 9 phenolic compounds, 7 flavonoids, 4 alkaloids, 4 tannins, and 4 terpenoids (Table 3; Fig. 2). Major phenolics were gallic acid (20.41 mg/g), ferulic acid (15.06 mg/g), and hydroxycinnamic acid (14.74 mg/g). Major flavonoids were chrysin (19.85 mg/g), rutin (14.80 mg/g), and kaempferol (10.12 mg/g). Alkaloids included atropine (13.88 µg/g), scopolamine (9.11 µg/g), and trigonelline (8.02 µg/g). Major tannins were ellagitannins (19.03 mg/g), proanthocyanidins (17.23 mg/g), and gallotannins (8.69 mg/g). Major terpenoids were linalool (18.02 mg/g), cymene (9.85 mg/g), and camphene (8.17 mg/g).

**Table 3:** HPLC analysis of *E. camaldulensis* extract

Class	Peak name	Concentration (mg/g)	RT (min)
Phenolic compounds	Gallic	20.41	11.0
	Ferulic	15.06	9.0
	Hydroxycinnamic	14.74	18.1
	Cinnamic	13.65	6.0
	Coumaric	12.17	13.0
	Vanillic	11.82	16.0
	Syringic acid	9.61	15.0
	Ellagic	6.74	8.0
	Chlorogenic	5.30	5.0
Flavonoids compounds	Chrysin	19.85	3.0
	Rutin	14.80	14.8
	Kaempferol	10.12	9.0
	Catechin	8.63	6.3
	Luteolin	6.74	12.8
	Apigenin	4.74	4.2
	Galangin	4.22	10.3
Alkaloids	Atropine	13.88	5.0
	Scopalamine	9.11	7.1
	Trigonelline	8.02	3.0
	Hyocyamine	6.36	9.8
Tannins	Ellagitannins	19.03	8.0
	Proanthocyanidins	17.23	3.5
	Gallotannins	8.69	10.1
	Tannic acid	5.69	13.3
Terpenoids	Linalol	18.02	3.0
	Cymene	9.85	6.7
	Camphene	8.17	5.0
	Pinene	7.69	8.0

### Effect of thalidomide and/or *E. *extract on Kasumi-1 cell viability

**Fig. 3:**
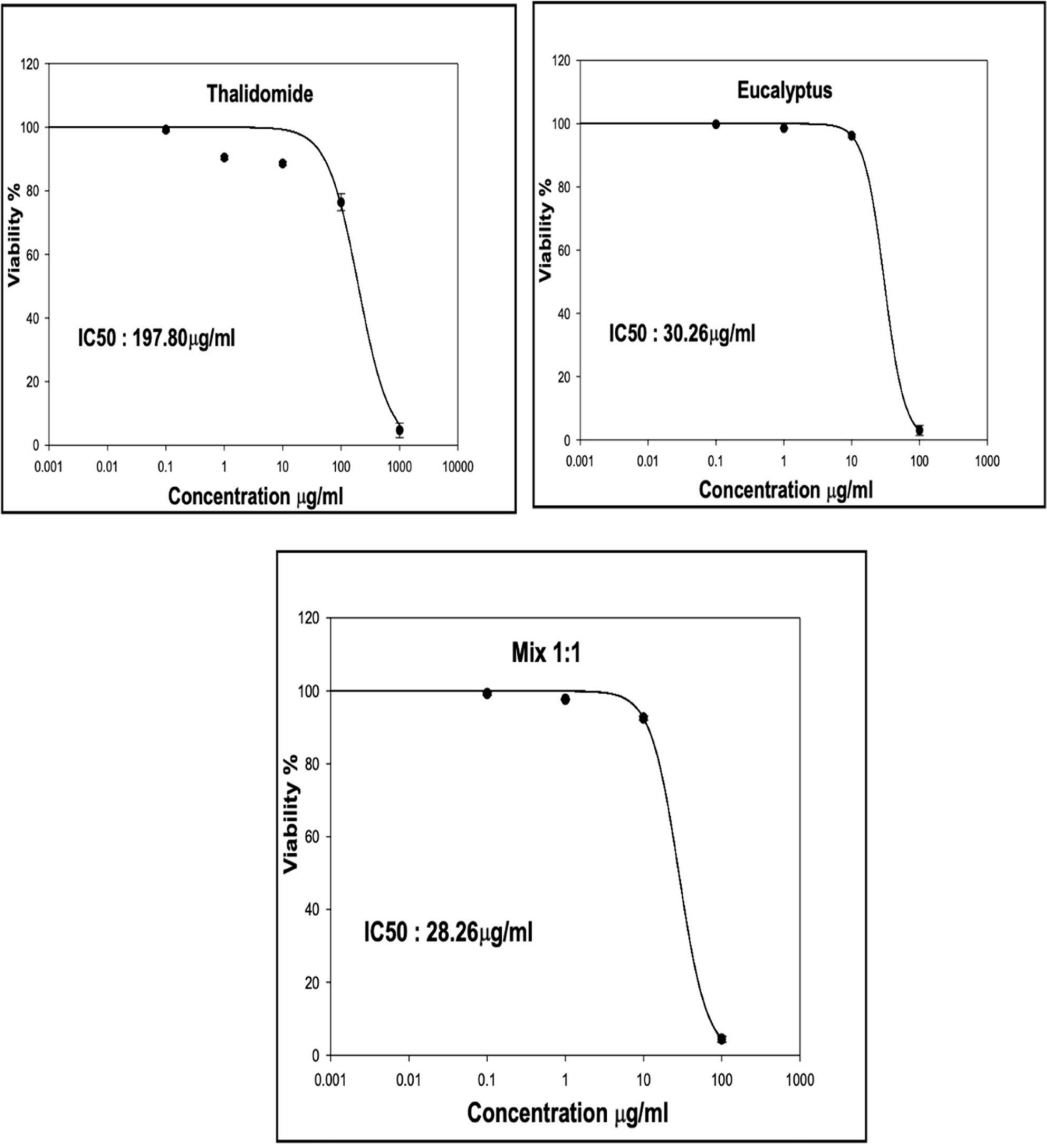
The effect of Thal (**A**), *E*. Extract (**B**) and the combination of Thal and *E.* Extract (**C**) on Kasumi-1 cell viability (%)

As illustrated in Fig. 3, mean cell viability (%) was 100% for untreated controls. For thalidomide-treated cells, viability was 99.22%, 90.43%, 88.57%, 76.38%, and 4.69% at 0.1, 1, 10, 100, and 1000 µg/mL, respectively. For extract-treated cells, viability was 99.78%, 98.56%, 96.16%, and 2.99% at 0.1, 1, 10, and 100 µg/mL, respectively. For combination-treated cells, viability was 99.24%, 97.66%, 92.53%, and 4.56% at 0.1, 1, 10, and 100 µg/mL, respectively.

IC₅₀ values were 197.80 µg/mL for thalidomide, 30.26 µg/mL for the extract, and 28.26 µg/mL for the combination. The combination index (CI) was 1, indicating an additive effect. Statistical analysis showed that treatment with thalidomide and/or extract significantly reduced cell viability compared with untreated controls in a concentration-dependent manner (*p* < 0.05).

### Effect on IL-6 gene expression

**Fig. 4:**
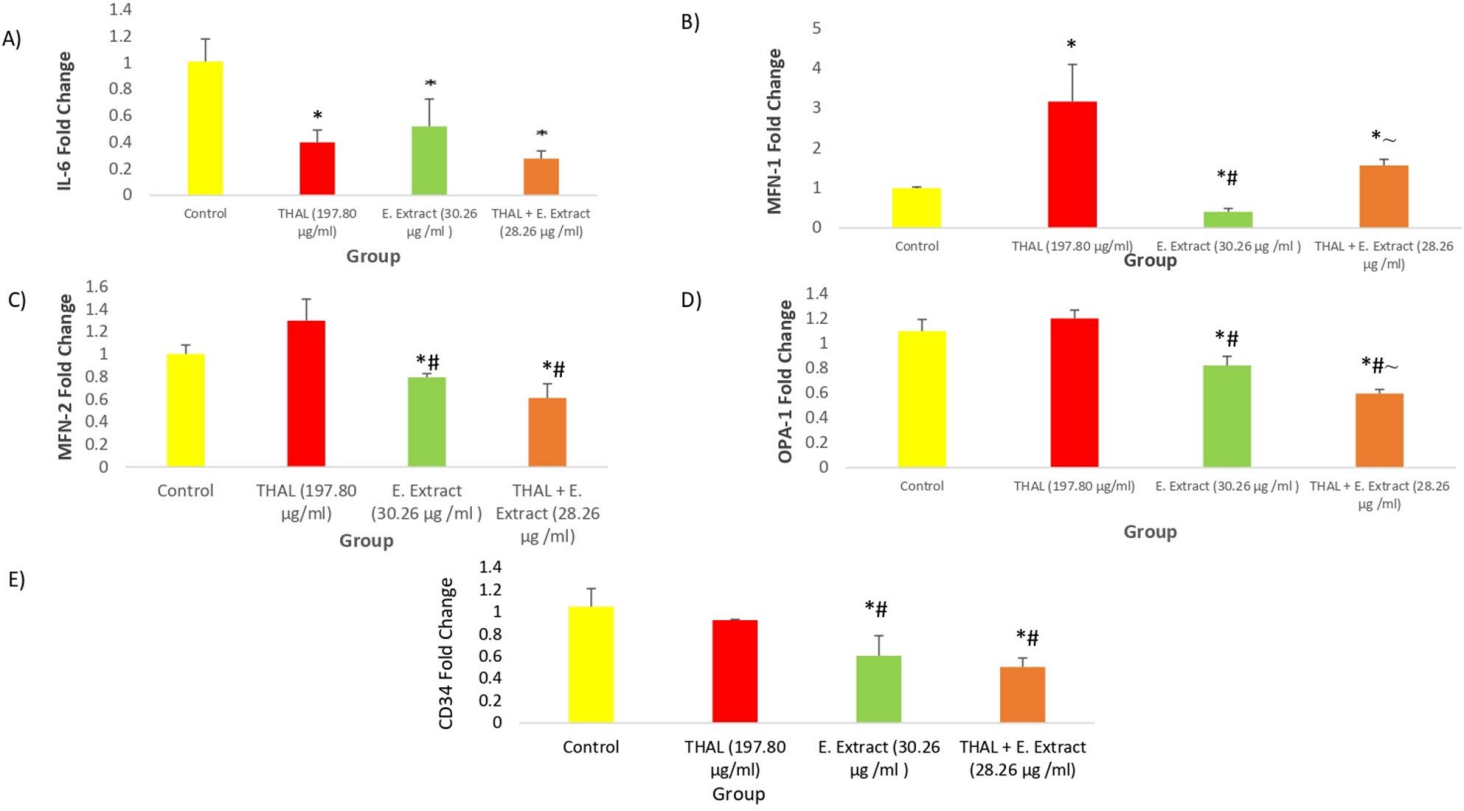
Effect of thalidomide and/or *Eucalyptus camaldulensis* extract on gene expression in Kasumi-1 cells. Relative mRNA expression levels of IL-6, MFN1, MFN2, OPA1, and CD34 quantified by qRT-PCR. Expression normalized to GAPDH. Data expressed as mean ± SD (*n* = 3). * *p* ≤ 0.05 vs. control; # *p* ≤ 0.05 vs. thalidomide-treated; ~ *p* ≤ 0.05 vs. extract-treated

As presented in Table 4; Fig. 4A, IL-6 expression was significantly reduced in thalidomide-, extract-, and combination-treated cells compared with controls (*p* = 0.006, *p* = 0.034, and *p* = 0.002, respectively). Differences between treatment groups were not statistically significant (thalidomide vs. extract *p* = 0.409; thalidomide vs. combination *p* = 0.124; extract vs. combination *p* = 0.121).

**Table 4:**
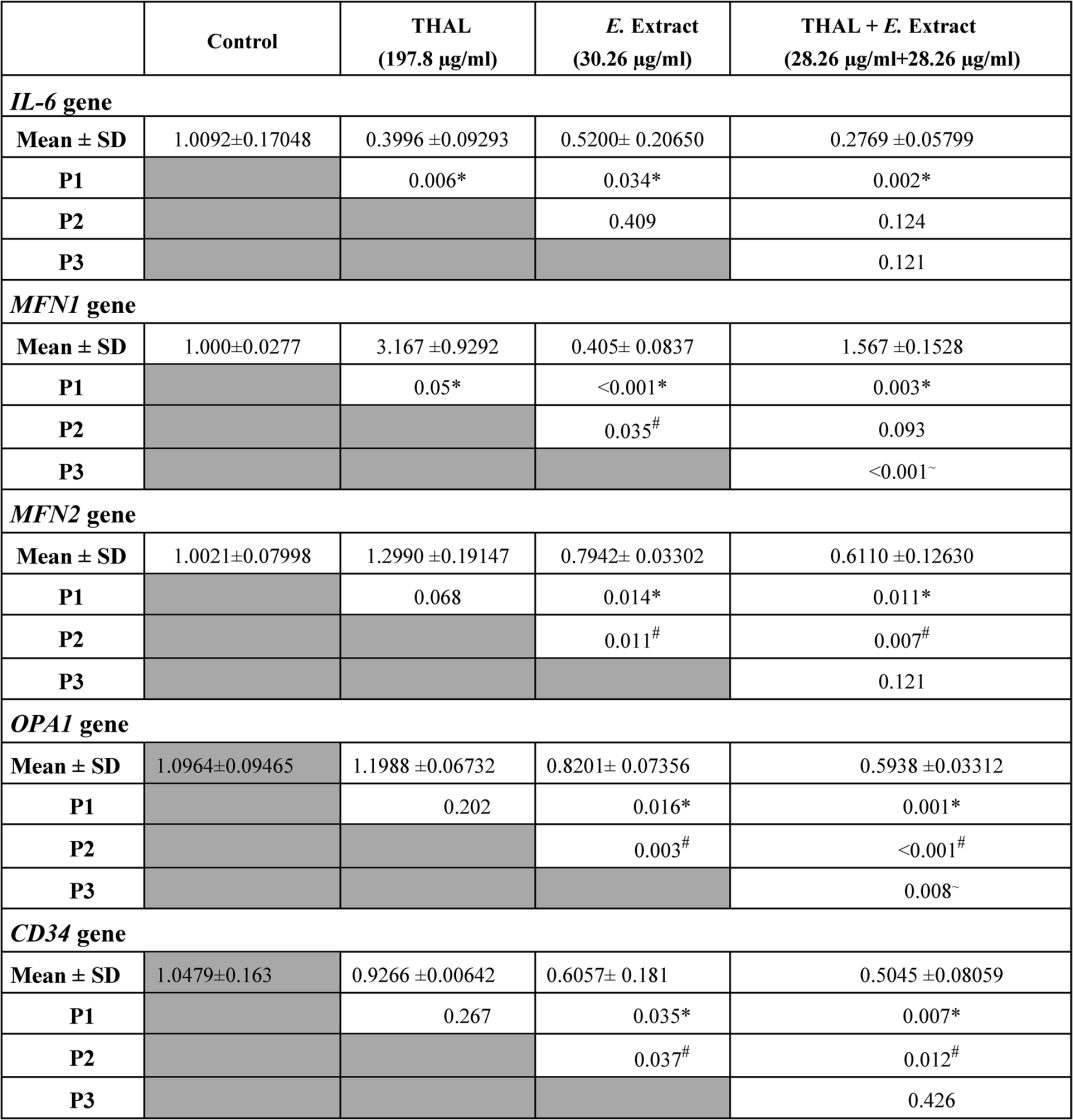
Statistical analysis of *IL-6*,* MFN1*,* MFN2*,* OPA1 and CD34* gene expression in all studied group (*n* = 3)

P1: P value for comparing between control group and the other studied groups

P2: P value for comparing between THAL group and *E.* Extract groups either alone or in combination with THAL

P3: P value for comparing between groups treated with *E*. Extract alone and in combination with THAL

* *p* ≤ 0.05 vs. control; # *p* ≤ 0.05 vs. thalidomide-treated; ~ *p* ≤ 0.05 vs. extract-treated

### Effect on MFN1 gene expression

As presented in Table 4; Fig. 4B, MFN1 expression was significantly higher in thalidomide-treated and combination-treated cells compared with controls (*p* = 0.05 and *p* = 0.003, respectively), while extract treatment significantly reduced MFN1 expression compared with controls (*p* < 0.001). MFN1 expression was significantly higher with thalidomide than with extract (*p* = 0.035) and insignificantly higher than in the combination group (*p* = 0.093). MFN1 expression differed significantly between extract and combination groups (*p* < 0.001).

### Effect on MFN2 gene expression

As presented in Table 4; Fig. 4C, MFN2 expression was insignificantly higher in thalidomide-treated cells compared with controls (*p* = 0.068). In contrast, MFN2 expression was significantly reduced in extract-treated and combination-treated cells compared with controls (*p* = 0.014 and *p* = 0.011, respectively). MFN2 expression was significantly higher with thalidomide than with extract (*p* = 0.011) or combination treatment (*p* = 0.007). The difference between extract and combination groups was not significant (*p* = 0.121).

### Effect on OPA1 gene expression

As presented in Table 4; Fig. 4D, OPA1 expression was insignificantly higher in thalidomide-treated cells compared with controls (*p* = 0.202). OPA1 expression was significantly reduced in extract-treated and combination-treated cells compared with controls (*p* = 0.016 and *p* = 0.001, respectively). OPA1 expression was significantly higher with thalidomide than with extract (*p* = 0.003) or combination treatment (*p* < 0.001). OPA1 expression differed significantly between extract and combination groups (*p* = 0.008).

### Effect on CD34 gene expression

As presented in Table 4; Fig. 4E, CD34 expression was insignificantly reduced by thalidomide compared with controls (*p* = 0.267). Extract and combination treatments significantly reduced CD34 expression compared with controls (*p* = 0.035 and *p* = 0.007, respectively). CD34 expression was significantly higher with thalidomide than with extract (*p* = 0.037) or combination treatment (*p* = 0.012). The difference between extract and combination groups was not significant (*p* = 0.426).

### Effect on ATP level

**Fig. 5:**
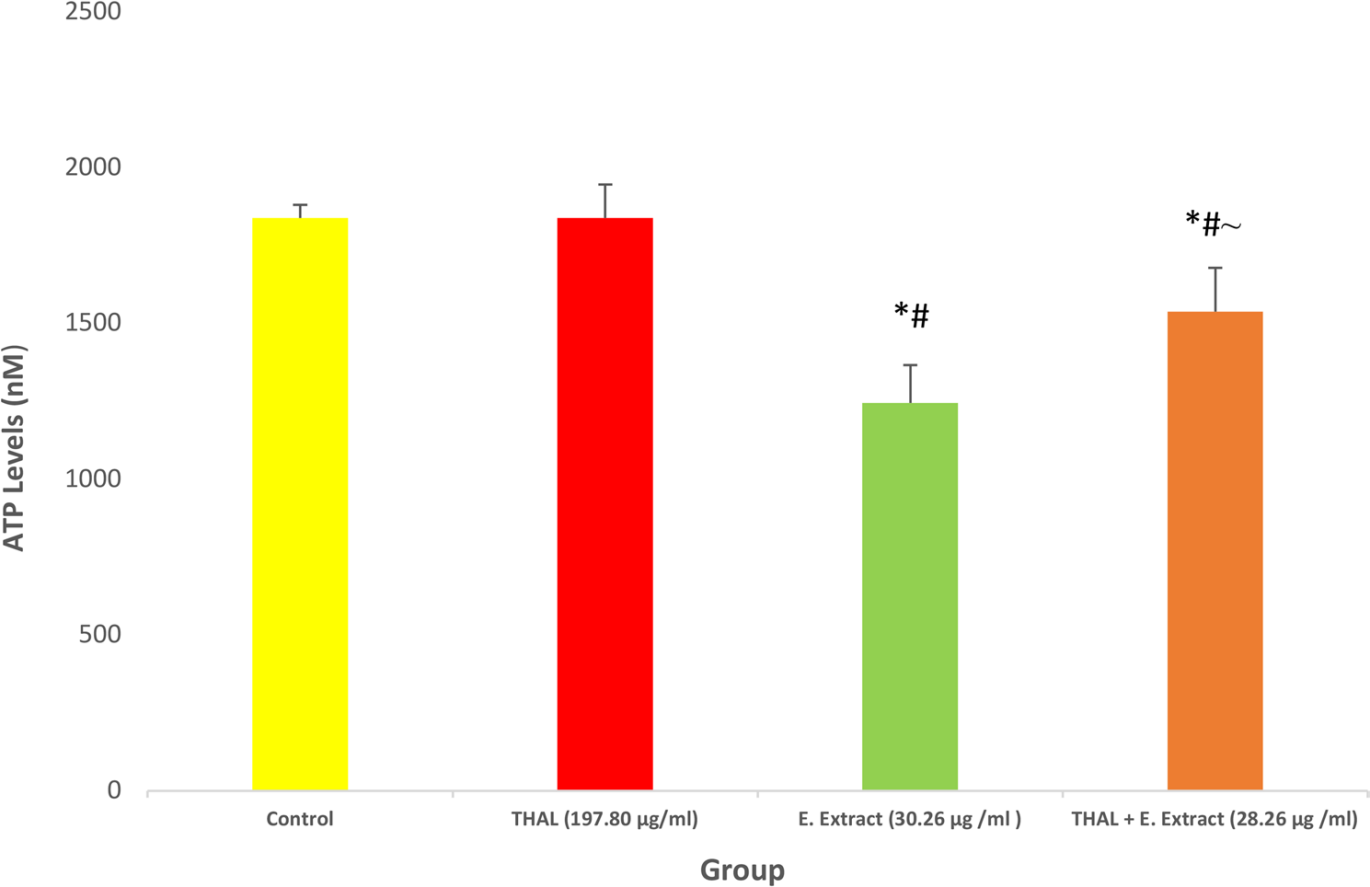
Effect of thalidomide and/or *Eucalyptus camaldulensis* extract on intracellular ATP levels. ATP concentration measured by ELISA after 48 h treatment. Data expressed as mean ± SD (n = 3). * p ≤ 0.05 vs. control; # p ≤ 0.05 vs. thalidomide-treated; ~ p ≤ 0.05 vs. extract-treated

As illustrated in Fig. 5, mean ATP level (nM) was 1893.67 in untreated cells, 1838.33 in thalidomide-treated cells (197.8 µg/mL), 1243.75 in extract-treated cells (30.26 µg/mL), and 1537.50 in combination-treated cells (28.26 µg/mL thalidomide + 28.26 µg/mL extract). ATP level was insignificantly lower with thalidomide compared with controls (*p* = 0.452), while ATP was significantly reduced with extract alone (*p* = 0.001) and combination treatment (*p* = 0.013). ATP was significantly higher with thalidomide than with extract (*p* = 0.003) or combination treatment (*p* = 0.042). The difference between extract and combination groups approached but did not reach significance (*p* = 0.052).

### Effect on mitochondrial length

**Fig. 6:**
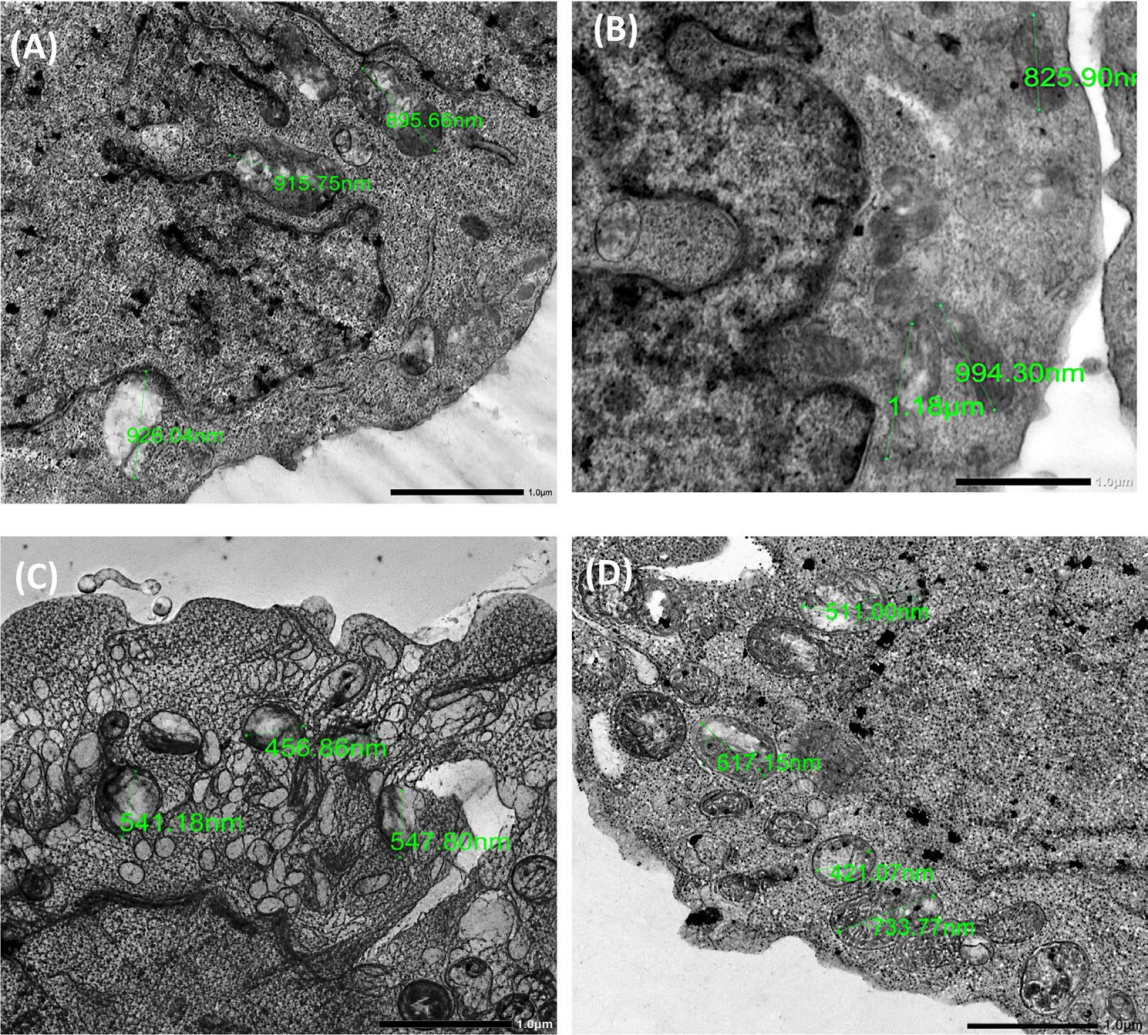
Representative transmission electron micrographs (×8000) of Kasumi-1 cells. (**A)** Control; (**B**) Thalidomide-treated (197.8 µg/mL); (**C**) *E*. Extract-treated (30.26 µg/mL); (**D**) Combination-treated. Arrows indicate mitochondria

**Fig. 7:**
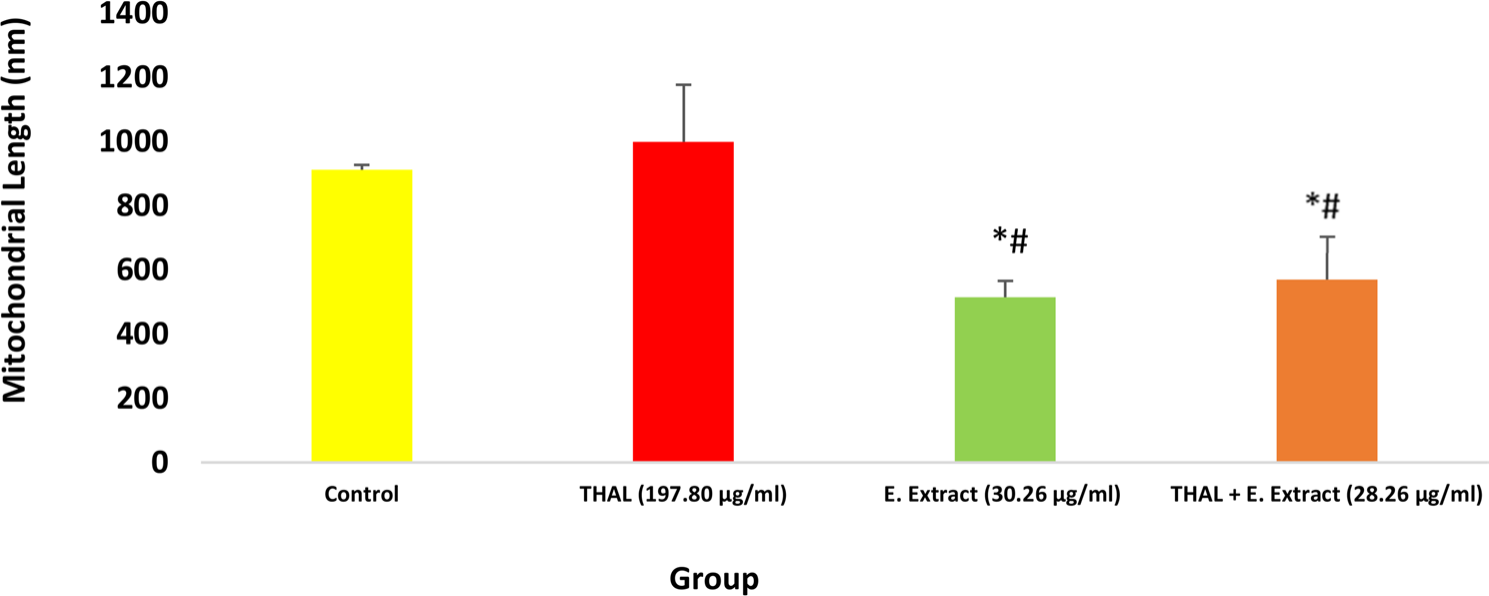
Effect of treatments on mitochondrial length in Kasumi-1 cells. Mean mitochondrial length (nm) measured from 20 mitochondria per group. Data expressed as mean ± SD (*n* = 3). * *p* ≤ 0.05 vs. control; # *p* ≤ 0.05 vs. thalidomide-treated

TEM analysis (Figs. 6 and 7) demonstrated variations in mitochondrial length across groups. Mean mitochondrial length (nm) was 912.48 in controls, 1000.07 in thalidomide-treated cells, 515.28 in extract-treated cells, and 570.75 in combination-treated cells. Mitochondrial length was insignificantly higher with thalidomide compared with controls (*p* = 0.442), while extract alone (*p* < 0.001) and combination treatment (*p* = 0.014) significantly reduced mitochondrial length compared with controls. Mitochondrial length was significantly higher with thalidomide than with extract (*p* = 0.01) or combination treatment (*p* = 0.014). The difference between extract and combination groups was not significant (*p* = 0.536).

## Discussion

In AML, oxidative phosphorylation plays a central role in maintaining LSC survival and therapy resistance [3, 5]. *E. camaldulensis* leaf ethanolic extract has been widely examined due to its antioxidant and anti-inflammatory properties and has been reported to contain biologically active compounds including terpenoids, tannins, flavonoids, polyphenolics, and alkaloids [24, 25]. To explore the plant’s pharmacological potential, we characterized phytochemical constituents in the ethanolic extract using GC–MS and HPLC, providing a basis to link chemical composition to observed anti-leukemic effects.

GC–MS analysis identified multiple compounds in *E. camaldulensis* extract. Several fatty acids and terpenoid-related compounds have been reported to possess anticancer activity [26–29]. These findings are consistent with prior phytochemical analyses of *E. camaldulensis* extracts [17].

HPLC analysis demonstrated that the extract contained phenolic acids, flavonoids, alkaloids, tannins, and terpenoids. Major phenolics and flavonoids such as gallic acid, rutin, and kaempferol. The Alkaloids in *E.* Extract are Atropine, Scopalamine and Trigonelline. The major tannin compounds are Ellagitannins, Proanthocyanidins and Gallotannins. The major terpenoids are Linalol, Cymene and Camphene. These compounds have been associated with cytotoxic, apoptosis-inducing, and anti-metastatic effects in cancer models [30–36]. Collectively, the presence of these compounds supports the premise that *E. camaldulensis* may act as a source of natural anticancer agents.

Regarding cytotoxicity, both thalidomide and *E. camaldulensis* extract reduced Kasumi-1 viability in a concentration-dependent manner. Thalidomide has been reported to inhibit Kasumi-1 proliferation via apoptotic pathways and modulation of survival regulators [37]. Similarly, *E. camaldulensis* extracts have reduced viability in leukemia and solid tumor cell lines through anti-proliferative and pro-apoptotic mechanisms [14, 38, 39]. Notably, combination treatment produced an additive effect (CI = 1), indicating that the extract enhanced thalidomide’s activity without antagonism [40].

IL-6 is implicated in AML cell survival and chemoresistance and promotes signal transducer and activator of transcription 3 (STAT3) signaling and mitochondrial adaptations [41–44]. In the current study, thalidomide, extract, and combination treatments significantly downregulated IL-6 expression. Thalidomide suppresses cytokine production through immunomodulatory mechanisms involving nuclear factor kappa B signaling [45]. Extracts from *Eucalyptus* species have also been associated with reduced IL-6 production in Lipopolysaccharide-induced polymorphonuclear cells and this effect may be due to the presence of gallic acid and other phenolic compounds [46].

Mitochondrial fusion proteins (MFN1, MFN2, and OPA1) regulate mitochondrial morphology and respiratory efficiency [7]. Enhanced mitochondrial fusion has been described as a therapeutic vulnerability in AML [8]. In this study, *E. camaldulensis* extract significantly downregulated MFN1, MFN2, and OPA1, consistent with impaired mitochondrial dynamics. Based on previous studies, this may be due to suppression of STAT3 as a result of downregulation of IL-6 by *E. Extract* either alone or in combination with THAL [43, 44, 47]. This finding supports the hypothesis that targeting IL-6 can disrupt mitochondrial dynamics and viability in AML cells, offering a potential therapeutic strategy to impair leukemic cells metabolism and promote cell death. Noteworthy, STAT3 expression must be determined in future studies to clarify the mechanism of mitochondrial fusion genes downregulation in the current study.

Extract treatment significantly reduced intracellular ATP levels and shortened mitochondrial length. Prior work suggests that perturbation of fusion regulators can decrease oxygen consumption and ATP generation [8, 48]. Mitochondrial elongation has been associated with enhanced OXPHOS during leukemic proliferation [49]. Thus, the decrease in the mitochondrial length by *E. Extract* either alone or in combination with THAL can be explained by downregulation of mitochondrial fusion genes as observed in the current study which may lead to metabolic shift toward glycolysis and subsequently decrease in ATP level.

LSCs exhibit high dependence on OXPHOS to fuel ATP-dependent drug efflux transporters [5, 6]. CD34 is commonly used to identify LSC-associated compartments in AML [9, 10]. In this study, *E. camaldulensis* extract significantly downregulated CD34 expression. Disruption of OXPHOS may reduce energy availability required for resistance mechanisms [5, 6, 50] .

The main limitations of this study, lack of normal cells like peripheral blood mononuclear cells (PBMCs) to establish *E. camaldulensis* leaf extract selectivity, it was conducted using a single AML cell line and did not include protein-level validation or direct mitochondrial respiration assays as well as mitochondrial fission. Future studies should evaluate these findings in additional AML models and in vivo systems and assess *E. camaldulensis* leaf extract selectivity using normal cells like PBMCs. Also, the chemo-sensitizing effect of *E. camaldulensis* leaf extract with standard therapeutic modalities in AML should be investigated in future studies.

Overall, the findings suggest that *E. camaldulensis* extract exerts in vitro anti-leukemic effects by disrupting mitochondrial energetics and downregulating an LSC-associated marker. The additive interaction with thalidomide supports further investigation as a complementary therapeutic strategy in AML.

## Conclusion

To the best of our knowledge, this is the first study demonstrating that *Eucalyptus camaldulensis* leaf extract, either alone or in combination with thalidomide, may target leukemic stem cell–associated pathways in AML through mitochondrial dysfunction consistent with reduced OXPHOS activity mediated by downregulating IL-6 and mitochondrial fusion genes. In contrast, thalidomide alone exerted limited effects on mitochondrial bioenergetics and LSC-associated marker. Overall, *E. camaldulensis* extract exhibited a stronger impact on mitochondrial metabolism than thalidomide and may represent a promising complementary therapeutic strategy to overcome metabolic-driven therapy resistance in AML. 

## Data Availability

The datasets generated and/or analyzed during the current study are available from the corresponding author upon reasonable request.

## Abbreviations

AML: Acute myeloid leukemia
LSCs: Leukemic stem cells
IL-6: Interleukin 6
MFN1: Mitofusin 1
MFN2: Mitofusin 2
OPA1: Optic atrophy 1
OXPHOS: Oxidative phosphorylation
ATP: Adenosine triphosphate
CD34: Cluster of Differentiation 34
GC-MS: Gas Chromatography-Mass Spectrometry
HPLC: High-Performance Liquid Chromatography
STAT3: Signal transducer and activator of transcription 3
PBMCs: Peripheral blood mononuclear cells
